# Multi-Objective Optimization for Nano-Silica-Modified Concrete Based on Explainable Machine Learning

**DOI:** 10.3390/nano15181423

**Published:** 2025-09-16

**Authors:** Yue Gu, Ruyan Fan, Yikun Li, Jiaqiang Zhao, Zijian Song, Hongqiang Chu

**Affiliations:** 1 College of Civil and Transportation Engineering, Hohai University, Nanjing 210098, China; 2 College of Materials and Science and Engineering, Hohai University, Changzhou 213000, China

**Keywords:** concrete, machine learning, NSGA-II algorithm, multi-objective optimization, XGBoost algorithm

## Abstract

Nano-silica modified concrete (NSC) has been widely applied in engineering practice. However, conventional manual mix proportion design is both time-consuming and costly. In this study, four machine learning models—XGBoost, CatBoost, Random Forest, and AdaBoost—were trained to predict the compressive strength of NSC. Based on the best-performing model, the NSGA-II algorithm was employed to develop a multi-objective optimization framework, considering compressive strength, cost, and carbon emissions as objectives. The results indicated that XGBoost achieved the highest accuracy, with R^2^ = 0.99 and RMSE = 1.80 MPa. Feature importance analysis further revealed that nano-silica content was strongly correlated with strength (0.82) and cost (0.85). Using NSGA-II, a set of Pareto-optimal solutions was generated. The NSGA-II algorithm produced Pareto-optimal solutions, highlighting the trade-offs among the three objectives. This integrated approach effectively reduces experimental workload and provides a valuable reference for sustainable NSC mix proportion design.

## 1. Research Background

Among the primary building materials, concrete exhibits the best sustainability value in terms of carbon emissions per unit mass compared to steel, glass, wood, and bricks. However, due to its massive usage, the total carbon emissions from concrete are the highest, accounting for approximately 7% of global carbon emissions. Enhancing the performance of concrete [1], such as improving its durability and reducing its carbon footprint, is crucial. Nanomodification is one of the key technological approaches for performance enhancement.

Nano-silica is the most commonly used nanoparticle in concrete. This is partly due to its economic feasibility, with a price point that is acceptable for most applications, and partly because it significantly improves various properties of concrete. Its effects include filling, nucleation, and pozzolanic effects. S. Poon [1] found that adding an appropriate amount of nano-silica to high-performance concrete can significantly increase its early (3-day) and late (28-day) compressive strength. Similarly, M. Behfarnia [2] noted that incorporating nano-silica enhances the early (within 7 days) compressive strength and elastic modulus of self-compacting concrete. Y. Li [3] discovered that the addition of nano-silica effectively reduces the chloride ion penetration rate in cement mortar, thereby improving its resistance to corrosion. S. Thomas [4] showed that using nano-silica as an additive not only decreases the rate of chloride ion penetration through concrete but also enhances its carbonation resistance, collectively extending the service life of structures. V. Mehta [5] highlighted that nano-silica not only improves multiple durability-related properties, such as wear resistance and freeze–thaw cycle resistance, but also promotes the formation of a denser microstructure, further enhancing overall performance.

In recent years, several studies have investigated the effects of nano-silica addition on specific properties of concrete composites. For example, Sun, M et al. [6] evaluated high-performance pervious concrete mixed with nano-silica and carbon fiber, reporting significant improvements in mechanical and permeability performance. Golewski et al. [7] determined fracture mechanic parameters of concretes based on cement matrix enhanced by fly ash and nano-silica, highlighting the role of nano-silica in improving fracture toughness. Other recent works have reported on mechanical, durability, and sustainability aspects of nano-silica modified concrete, as well as optimization approaches for its mix design.

Traditional concrete design has primarily focused on optimizing mechanical properties. However, with the growing emphasis on reducing carbon footprints and controlling costs, driven by stricter government regulations and buyer demands, there is a shift toward multi-objective concrete design, aiming for a more balanced and sustainable optimization approach.

Machine learning (ML) techniques have been widely applied to various engineering problems, and their use in solving multi-objective mix proportion design for concrete has gained attention from scholars in the field of building materials. Zhang et al. [8] used a multi-objective particle swarm optimization algorithm to optimize the mix proportions of high-performance concrete, addressing dual-objective and triple-objective optimization problems for plastic concrete. Ren [9] developed an optimization model for high-performance concrete, using mix proportions as design variables and compressive strength, unit cost, and carbon emissions as optimization objectives. A solution method based on random forest algorithms and adaptive evolutionary multi-objective particle swarm optimization (AEPSO) was proposed, and the AEPSO algorithm was used to iteratively search for the optimal Pareto front. On one hand, artificial intelligence algorithms are popular due to their ability to quickly and accurately uncover intrinsic data relationships. On the other hand, metaheuristic search techniques, such as the non-dominated sorting genetic algorithm II (NSGA-II), are favored for their strong global search capabilities and ease of implementation in solving concrete mix proportion optimization problems. Fan [10] combined a gradient boosting prediction model with the NSGA-II algorithm to perform multi-objective optimization of recycled aggregate concrete (RAC) mix proportions, optimizing key objectives such as cost, carbon emissions, and compressive strength. The algorithm successfully obtained the Pareto front for the RAC three-objective optimization problem, providing guidance for RAC preparation. Using the NSGA-II algorithm to solve multi-objective mix proportion design problems for concrete not only yields mix proportions that meet all performance criteria but also improves work efficiency. Although research on multi-objective algorithms in concrete has gradually expanded, studies on performance prediction models and mix proportion optimization design for NSC are still limited. Unlike previous studies applying similar XGBoost–NSGA-II frameworks to other types of concrete, this study specifically addresses nano-modified concrete by simultaneously optimizing compressive strength, cost, and carbon emissions. The integrated framework not only predicts the compressive strength accurately for different curing ages but also incorporates life-cycle cost and carbon footprint analysis, enabling sustainable and economically viable mix design decisions for NSC.

**Figure 1:**
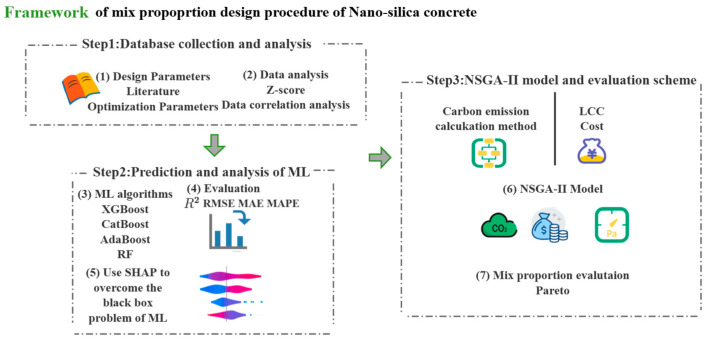
Framework of NSC mix design procedure.

This study proposes a novel method for the multi-objective optimization of nano-silica concrete (NSC) mix proportions, focusing on carbon emissions, cost, and compressive strength, using machine learning techniques. The research utilizes several algorithms, including XGBoost (eXtreme Gradient Boosting), CatBoost (Categorical Boosting), Random Forest (RF), AdaBoost (Adaptive Boosting), and NSGA-II, to establish a nonlinear relationship between NSC performance and material quantities, facilitating intelligent multi-objective optimization. First, an extensive database is constructed, containing NSC mix proportion data and compressive strength test results. Next, four machine learning algorithms—XGBoost, CatBoost, RF, and AdaBoost—are applied to model the relationships between NSC compressive strength, carbon emissions, cost, and the quantities of each constituent material. The predictive accuracy of these models is compared, and the most accurate model is selected for the compressive strength prediction. A mathematical model is then developed with compressive strength, cost, and carbon emissions as optimization objectives. The NSGA-II algorithm is employed to solve for the Pareto optimal solutions, and the optimal mix proportions are identified based on various preference criteria. Figure 1 shows the overall framework of this study.

## 2. Proportioning Database

By retrieving relevant literature and extracting useful data, a database was established. The compressive strength data of NSC includes 233 samples from existing literature [11,12,13,14,15,16,17,18,19,20,21,22,23,24,25], which were expanded to 699 samples using the CatBoost algorithm. This increase primarily resulted from the completion of strength–age pairs. The method enriched the dataset, reduced sparsity, and improved the robustness of the machine learning models. Except for the narrow range of high-efficiency water reducer values, other parameters vary significantly between their maximum and minimum values. This demonstrates the broad scope of the database, which is beneficial for improving the applicability of the established machine learning models. The ranges of each component’s content are explained as shown in Table 1.

From Table 1, it can be observed that the input range for cement is 80–630 kg/m^3^, 0–685 kg/m^3^ for fly ash, 0–1134 kg/m^3^ for slag, 0–60 kg/m^3^ for nano-silica, 0–60 kg/m^3^ for silica fume (used in some cases), 90–1007.55 kg/m^3^ for water, 0–21.38 kg/m^3^ for high-efficiency water reducer, 654–1278 kg/m^3^ for coarse aggregate, and 472–9320 kg/m^3^ for fine aggregate. In terms of output parameters, the compressive strength increases with higher cement content and decreases with higher water content, ranging from 2.995 to 95.25 MPa. Additionally, higher strength is associated with greater carbon emissions.

**Figure 2:**
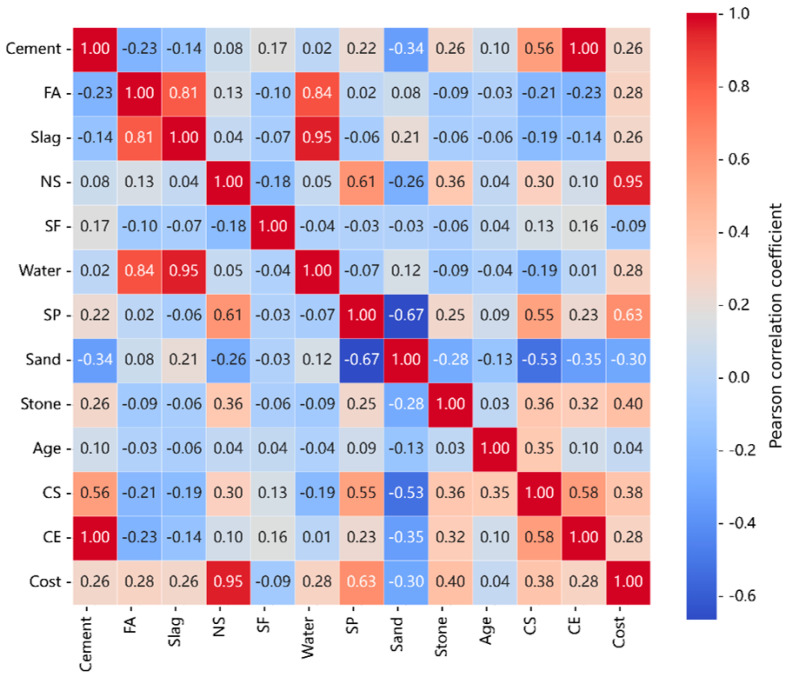
Heatmap of input and output variable correlations.

The correlation matrix represents the correlation coefficients between input and output variables, indicating positive or negative relationships between each variable. As shown in Figure 2, the results demonstrate the following: For compressive strength, nano-silica (kg/m^3^) has the highest correlation coefficient (0.82), indicating that nano-silica has the greatest impact on compressive strength. For carbon emissions, nano-silica (kg/m^3^) has the highest correlation coefficient (0.78), indicating that nano-silica has the greatest impact on carbon emissions. For cost, nano-silica (kg/m^3^) has the highest correlation coefficient (0.85), indicating that nano-silica has the greatest impact on cost.

## 3. Carbon Emission Calculation Method

For carbon emission calculation methods, there are clear standards both domestically and internationally, all of which are based on the perspective of the product’s full life cycle. For example, PAS 2050, also known as the “Specification for the Assessment of the Life Cycle Greenhouse Gas Emissions of Goods and Services,” was developed by the British Standards Institution (BSI). This specification defines the calculation boundaries for product carbon emissions, encompassing stages such as product design, production, usage, and transportation [26]. The ISO 14040 “Environmental Management—Life Cycle Assessment—Principles and Framework” and ISO 14044 “Environmental Management—Life Cycle Assessment—Requirements and Guidelines,” published by ISO in 2006, specify the scope of carbon emission accounting for product life cycles, the determination of functional boundaries, and the definition of reference flows [27,28]. Currently, China adopts the GB/T 24040-2008 “Environmental Management—Life Cycle Assessment—Principles and Framework” and GB/T 24044-2008 “Environmental Management—Life Cycle Assessment—Requirements and Guidelines,” which are equivalent conversions of these international standards [29,30].

The above standards provide macro-level regulations on the carbon emission calculation boundaries for the full life cycle of products or services, offering significant guidance for calculating carbon emissions over the life cycle of building materials.

Although scholars both domestically and internationally have produced some research results, their focuses vary. Some scholars emphasize the impact of concrete material selection and mix proportions on carbon emissions. For example, Ellis Gartner [31] proposed using hydraulic cement as a substitute for traditional Portland cement and incorporating industrial by-products such as fly ash and slag, or natural pozzolanic materials, into concrete to reduce carbon emissions. Webster et al. [32] used the BEE 4.0 assessment software developed by NTIS to calculate the carbon emissions of C25-grade concrete beams, finding that replacing 50% of cement with slag reduced carbon emissions by 26%. Other studies focus on carbon emissions caused by the transportation of concrete materials. For instance, Alma Artenian et al. [33] developed a GIS-based model to reduce carbon emissions from transportation by optimizing parameters such as transportation routes. Additionally, some research considers multiple stages of the concrete product formation process for carbon emission analysis. For example, Radlinski et al. [34] considered carbon footprints across various stages, including raw material production, concrete material production, transportation, construction, maintenance, and demolition/recycling of concrete structures, but did not provide detailed calculation methods or bases. Dias et al. [35] considered carbon emissions from energy consumption during concrete production, embodied carbon in raw materials, and input energy, calculating the carbon (CO_2_) emissions of concrete to be 312 kg/m^3^, but did not account for the transportation of raw materials and products. Gao et al. [36] considered the embodied carbon of raw materials and the carbon emissions from concrete production and transportation, calculating the carbon emissions (CO_2_) of conventional C30 concrete to be 187.84 kg/m^3^, but did not include carbon emissions from raw material transportation. Regardless of the research, whether domestic or international, different calculation boundaries lead to varying results in concrete carbon emission calculations.

**Figure 3:**
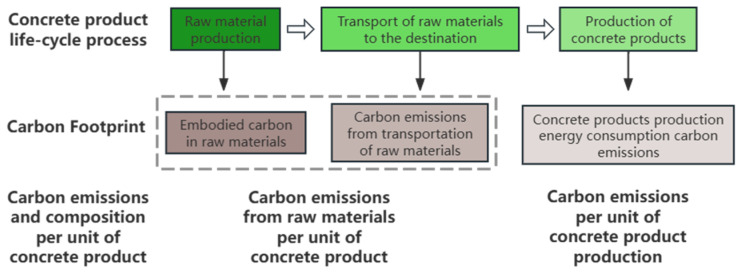
Carbon emission calculation boundary for nano-modified concrete products based on LCA (Life Cycle Assessment).

The calculation boundary of this model starts from the production of raw materials and ends at the production of the concrete product, aiming to calculate the carbon emissions per unit of nano-modified concrete product within this life cycle. Within this calculation boundary, three stages are included: raw material production, raw material transportation, and concrete production. The sources of carbon emissions consist of three parts: embodied carbon in raw materials, carbon emissions from raw material transportation, and carbon emissions from energy consumption during concrete production. The carbon emissions per unit of concrete are composed of the embodied carbon from raw materials and the carbon emissions from concrete production, as illustrated in Figure 3.

### 3.1. Carbon Emission Quantification Algorithm for Nano-Modified Concrete Products

Based on the above requirements for setting carbon emission boundaries and the analysis of carbon emission sources, the carbon emissions per unit of concrete product can be calculated using Formula (1) [37]:

(1) $$C=C_{Y}+C_{N}$$

C—Unit carbon emissions of concrete;

$C_{Y}$—Embodied carbon emissions from raw materials of concrete;

$C_{N}$—Carbon emissions from energy consumption during concrete production.

#### 3.1.1. Algorithm for Quantifying Embodied Carbon Emissions from Raw Materials per Unit of Concrete Product ($C_{Y}$) 

Step 1: Calculate the embodied carbon emissions per unit of raw material using Formula (2) [37]:

(2) $$EF_{i}=EF_{i,h}+EF_{i,y}$$

${EF}_{i}$—Embodied carbon emissions per unit of type i raw material;

${EF}_{i,h}$—Embedded carbon emissions per unit of type i raw material;

${EF}_{i,y}$—Transportation carbon emissions per unit of type i raw material.

${EF}_{i,y}$ can be calculated using Formula (3) [35]:

(3) $${EF}_{i,y}=E_{i,T}\cdot EF_{i,B}\cdot H_{i}$$

$E_{i,T}$—Unit energy consumption for the transportation mode of type i raw material (in Table 2);

$EF_{i,B}$—Effective CO_2_ emission factor for fuel combustion in the transportation of type i raw material (in Table 3);

$H_{i}$—Transportation distance for type i raw material.

**Table 2:** Unit energy consumption of transportation modes [38].

Transportation Mode	Unit Transportation Energy Consumption/MJ (t·Km)^−1^
Road transportation (Gasoline vehicle)	2.055

**Table 3:** Effective CO_2_ emission factor for automotive gasoline [37].

Fuel Type	Effective CO_2_ Emission Factor/(Kg·TJ^−1^)
Automotive gasoline	74,100

Based on the mix proportion of nano-silica-modified concrete, calculate the embodied carbon emissions from raw materials per unit of concrete product using the formula [37]:

(4) $$C_{Y}=\sum_{i=1}^{n}EF_{i}\cdot M_{i}\;$$

$M_{i}$—Amount of type i raw material per unit of concrete product

#### 3.1.2. Amount of Type i Raw Material per Unit of Concrete Product ($C_{N}$) 

The primary energy consumed during the production of concrete products is electricity, and the production energy consumption per unit product is calculated using Formula (5) [37]:

(5) $$C_{N}=\frac{E_{d}\cdot K_{d}}{N}$$

$E_{d}$—Total electricity consumption;

$K_{d}$—CO_2_ emission factor for electricity (taken as 0.7703 kg/kWh [37]);

N—Total production of concrete products.

### 3.2. Calculation Parameters for Carbon Emissions of Nano-Silica Modified Concrete Products

Based on the above concrete carbon emission calculation model, determining the carbon emissions of produced concrete products requires obtaining a series of parameters, as shown in Table 4, Table 5 and Table 6.

It is important to emphasize that the carbon emissions are for reference only. The purpose is to demonstrate the effectiveness of the calculation method used in this study. Due to differences in regions and time, there may be significant variations.

## 4. Introduction to Main Algorithms and Specific Implementation Methods

### 4.1. Main Algorithms

#### 4.1.1. AdaBoost Algorithm

AdaBoost (Adaptive Boosting) is an ensemble learning algorithm that constructs a strong classifier by combining multiple weak classifiers. It operates through iterative training, adjusting sample weights in each iteration so that samples misclassified in previous iterations receive more attention in subsequent iterations. This process gradually improves the overall classification performance.

#### 4.1.2. XGBoost Algorithm

The XGBoost algorithm was proposed by Chen et al. [40] in 2016 as an ensemble learning algorithm based on decision trees. It is one of the commonly used models in the field of machine learning. The algorithm serially trains individual decision trees to correct model errors, ultimately producing a more accurate and reliable prediction result.

#### 4.1.3. Random Forest Algorithm

The RF algorithm was proposed by Breiman [41] in 2001 as an ensemble learning algorithm based on bagging. This algorithm constructs multiple classification trees by performing bootstrap sampling on the original data and outputs the final result through voting or averaging their predictions. The model demonstrates excellent performance in both classification and regression problems, especially when handling high-dimensional data.

#### 4.1.4. CatBoost Algorithm

CatBoost (Categorical Boosting) is a gradient boosting decision tree algorithm particularly suited for handling categorical features. It improves model performance by automatically processing categorical variables, reducing overfitting, and using ordered boosting techniques. CatBoost excels in processing large-scale datasets and high-cardinality categorical features, making it widely applicable to various machine learning tasks.

#### 4.1.5. NSGA-II Algorithm

**Figure 4:**
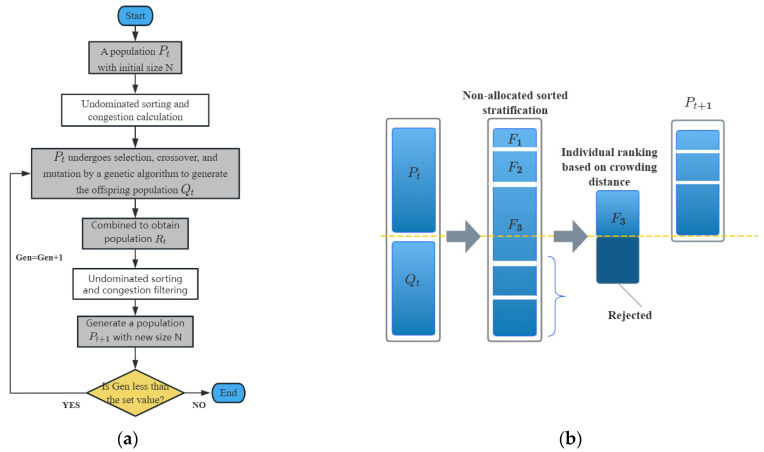
NSGA-II flowchart and optimization process diagram: (**a**) Flowchart; (**b**) Optimization process diagram.

NSGA-II is an enhanced genetic algorithm used for multi-objective optimization, rooted in the principle of Pareto optimality. The algorithm employs a fast non-dominated sorting approach and is characterized by its fast execution speed and good convergence properties. The principles of NSGA-II have been extensively explained in the literature [42,43,44]. The optimization process of the NSGA-II algorithm is shown in Figure 4 and mainly includes the following steps:

(1) Generate an initial population P_t_ of size N;
(2) Perform non-dominated sorting and crowding distance calculation on populationP_t_, followed by selection, crossover, and mutation operations to produce an offspring population Q_t_ of the same size as population P_t_ Finally, merge population P_t_ and population Q_t_ to create a new population R_t_ = {P_t_,Q_t_} with a total size of 2N.

Analyze the dominance relationships among all individuals within population R_t_ and perform hierarchical and rank sorting F_1_ = rank_1_ > ⋯ > F_n_ = rank_n_.

Based on the hierarchical sorting results from step (3), sequentially store individuals from higher to lower levels into the next-generation population P_(t+1)_. Simultaneously, reduce the population size from 2N to N. For levels that cannot be fully retained, sort and select individuals based on crowding distance. The optimization process is illustrated in Figure 4.

Repeat steps (2) to (4) until the specified number of iterations is reached or the convergence accuracy requirements are met. Finally, output the Pareto optimal solution set.

### 4.2. Specific Implementation Methods

**Figure 5:**
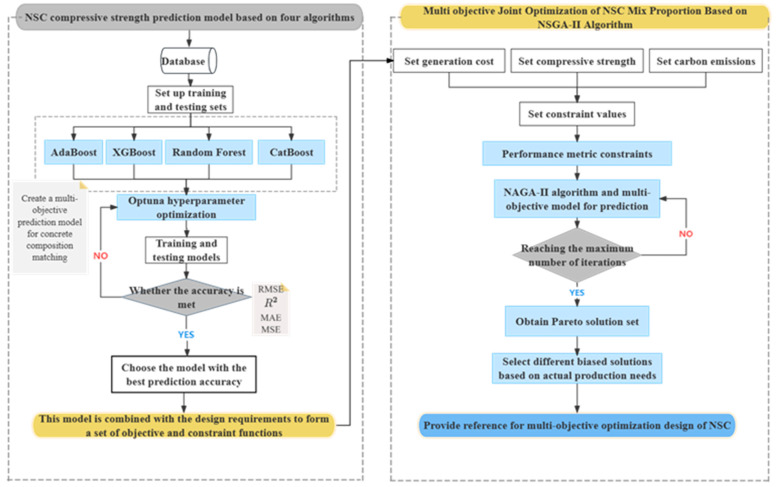
Implementation method diagram.

The specific implementation method of this study is divided into two modules. Module 1 first determines the algorithm through the database and then incorporates the NSGA-II optimization algorithm in Module 2, as shown in Figure 5.

### 4.3. Model Establishment

#### 4.3.1. Data Acquisition and Preprocessing

This study establishes a database by collecting mix proportions and performance test data of NSC. The dataset is preprocessed using normalization methods to eliminate the impact of different dimensions among the data on the model.

#### 4.3.2. Model Testing

Based on literature and practical engineering experience, the input parameters selected include curing age, compressive strength, cost, and carbon emissions. The output parameters are the concrete mix proportions, which include the amounts of cement, fly ash, slag, nano-silica, silica fume, water, high-efficiency water reducer, coarse aggregate, and fine aggregate.

The RMSE (Root Mean Square Error) is used to evaluate the global absolute deviation of the prediction results, while the coefficient of determination (R-square, R^2^) is used to measure the overall effectiveness of the model’s predictions. MAE (Mean Absolute Error) and MAPE (Mean Absolute Percentage Error) primarily reflect the degree of prediction error. The specific calculations are shown in the following formulas. The smaller the values of MAE, MAPE, and RMSE, and the closer R^2^ is to 1, the better the predictive performance of the model.

(6) $$RMSE=\sqrt{\frac{\sum_{i=1}^{n}{\left(y_{test}^{\left(i\right)}-y_{pred}^{\left(i\right)}\right)}^{2}}{n}}$$

(7) $$R^{2}=1-\frac{\sum_{i=1}^{n}{\left(y_{test}^{\left(i\right)}-y_{pred}^{\left(i\right)}\right)}^{2}}{\sum_{i=1}^{n}{\left(y_{test}^{\left(i\right)}-\bar{y_{test}}\right)}^{2}}$$

(8) $$MAE=\frac{\sum_{i=1}^{n}\left|y_{test}^{\left(i\right)}-y_{pred}^{\left(i\right)}\right|}{n}$$

(9) $$MAPE=\frac{1}{n}\sum_{i=1}^{n}\left|\frac{y_{test}^{\left(i\right)}-y_{pred}^{\left(i\right)}}{y_{test}^{\left(i\right)}}\right|$$

In the formula, $y_{test}^{\left(i\right)}$ represents the true value of the i-th sample, $y_{pred}^{\left(i\right)}$ represents the predicted value of the i-th sample, n represents the number of samples.

**Figure 6:**
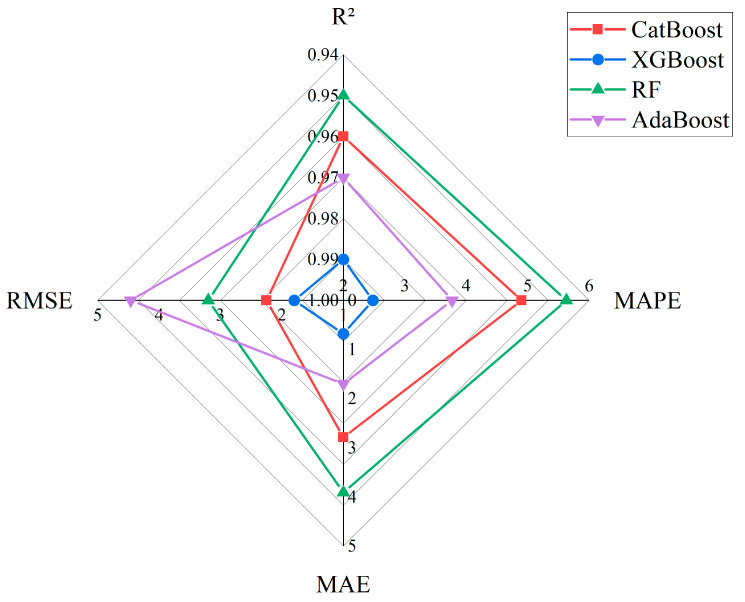
Schematic diagram of machine learning model performance metrics based on the dataset.

Table 7 provides the statistical results of the ML model performance. Additionally, to further demonstrate the effectiveness of the developed models, the performance metrics of each model are plotted separately in Figure 6. It can be observed that XGBoost (XGB) performs the best, with the highest R^2^ value of 0.99, and the lowest RMSE, MAE, and MAPE values of 1.8 MPa, 0.68 MPa, and 2.48%, respectively.

**Figure 7:**
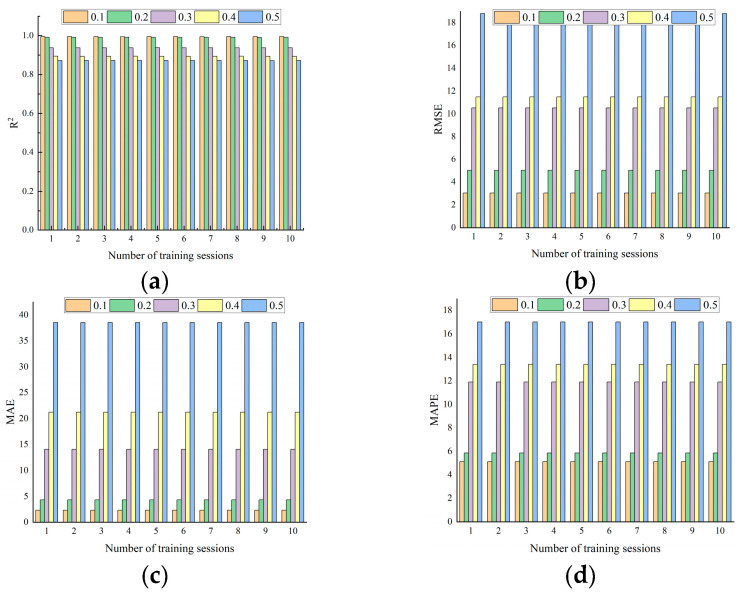
XGBoost model evaluation indicators: (**a**) R^2^; (**b**) RMSE; (**c**) MAE; (**d**) MAPE.

Figure 7 shows the results of training the model ten times based on different ratios of dividing the training and validation sets. It can be observed that the 9:1 division strategy achieves the closest R^2^ value to 1 and the smallest RMSE, MAE, and MAPE values across the ten training sessions. Therefore, by comparing the performance of the model evaluation metrics (R^2^, RMSE, MAE, and MAPE), the dataset is ultimately divided in a 9:1 ratio for model training and validation, respectively. The XGBoost machine learning model is employed to construct prediction models for concrete compressive strength, cost, and carbon emissions in relation to material mix proportions. Additionally, the Bayesian optimization method implemented in the Optuna library is used.

The process of hyperparameter tuning in Python3.9 typically involves using methods such as Grid Search, Random Search, or Bayesian Optimization to systematically explore and optimize the hyperparameters of a model. In this article, the Optuna framework is employed. Optuna is an advanced hyperparameter optimization framework that utilizes techniques like Bayesian Optimization and Tree-structured Parzen Estimator (TPE) to efficiently perform hyperparameter tuning. The tuning process of Optuna can be summarized into the following steps:

(1) Use Optuna’s trial object to dynamically generate hyperparameters. The objective function returns the model’s performance metric, RMSE, and Optuna attempts to minimize this metric;
(2) Create a Study object and specify the optimization direction (minimization);
(3) Call the study.optimize() method to begin the optimization and obtain the best-performing hyperparameter combination.

**Figure 8:**
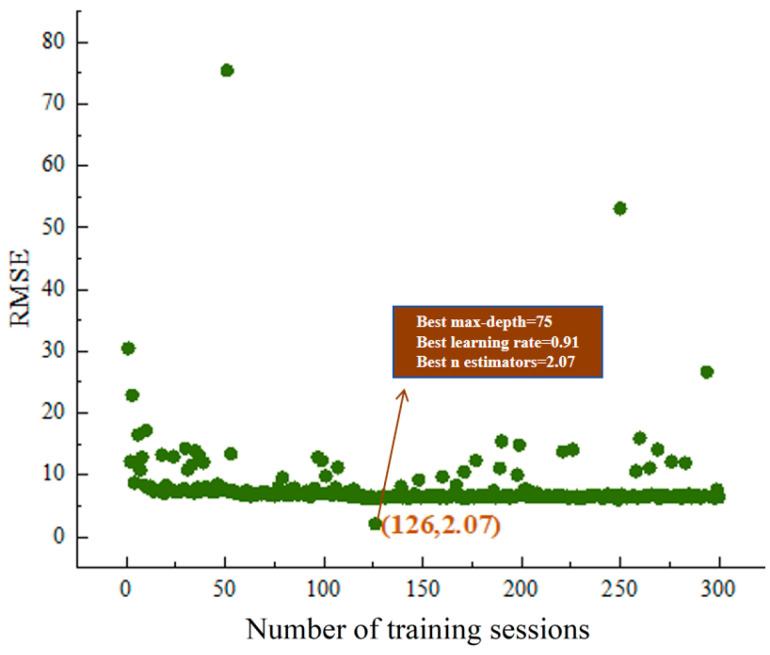
Multi-objective optimization of compressive strength, cost, and carbon emissions for the NSC mix ratio through XGBoost hyperparameter tuning.

The multi-objective optimization process of compressive strength, cost, and carbon emissions under XGBoost hyperparameter tuning is illustrated in Figure 8.

#### 4.3.3. Model Training

(1) Establish target values
  Set the target values as the compressive strength at the corresponding age, cost per cubic meter of concrete, and carbon emissions for NSC. There is a linear relationship between the production cost and carbon emissions per cubic meter of concrete and the mix proportion parameters. Specifically, target values $f_{1}$, $f_{2}$, $f_{3}$, $f_{4}$ represent compressive strength, production cost per cubic meter of concrete, carbon emissions, and age, respectively.
(2) Constraint conditions
  $$f_{1}\geq maxf_{1}$$
  $$f_{2}\leq minf_{2}$$
  $$f_{3}\leq minf_{3}$$
  $$f_{4}=f_{4}$$
(3) Multi-Objective optimization
  Based on the established objective functions and constraint conditions, the NSGA-II algorithm is employed to solve this mathematical model. Ultimately, the Pareto optimal front is obtained, which simultaneously satisfies the requirements for concrete strength at the specified age, cost, and carbon emissions.

## 5. Results

### 5.1. Engineering Case

**Figure 9:**
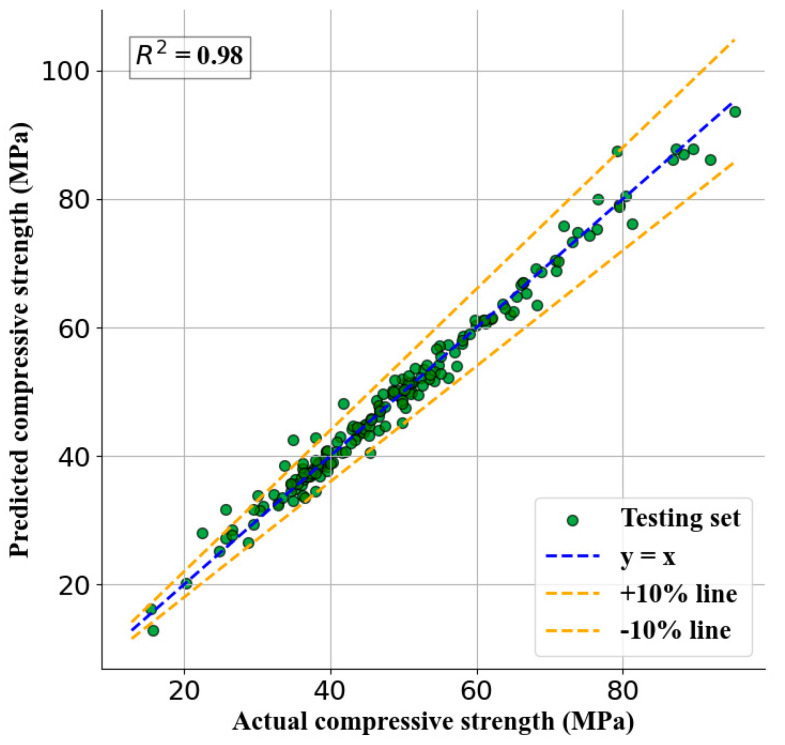
Correlation between actual compressive strength and predicted compressive strength.

By combining a specific engineering case, the feasibility and effectiveness of the proposed intelligent optimization design method for NSC mix proportions in this study are validated. The design requirements for the C30 nano-silica modified concrete needed for this project in Nanjing are as follows: the 28-day design strength should be higher than 30 MPa, the production cost per cubic meter should be 335 yuan, and the carbon emissions per cubic meter should be less than 188 kg. Since the sources of raw materials are stable (i.e., suppliers and material yards are relatively fixed), their quality characteristics can be considered essentially unchanged, and only the impact of usage needs to be considered. Based on the above information, the NSC30 mix proportion is optimized according to the design process outlined in this study. Figure 9 shows the fitting metrics between the predicted and actual values of the mix proportion data. The horizontal axis represents the experimental mix proportion data, while the vertical axis represents the predicted data from each model. The blue dashed line represents the equivalence line, and the closer the data points are to this line, the better the model’s fitting performance. Figure 9 illustrates the correlation between the predicted and actual values for all samples in the XGB model. In the figure, the green dots represent the dataset, and the blue diagonal dashed line with a slope of 1.0 indicates 100% prediction accuracy. The two dashed lines with slopes of 1.1 and 0.9, respectively, define a region representing a prediction error of ±10%. Most data points in the XGB algorithm model fall within the ±10% prediction error range, indicating superior prediction performance.

### 5.2. SHAP Interpretability

Due to the black-box problem, the application of ML models is limited. Feature importance analysis can effectively evaluate the impact of design variables on the compressive strength, carbon emissions, and cost of NSC. Therefore, the SHAP(SHapley Additive exPlanations) method is used to analyze the trained XGBoost model, which interprets the ML model by assessing the influence of design variables on the output variables.

**Figure 10:**
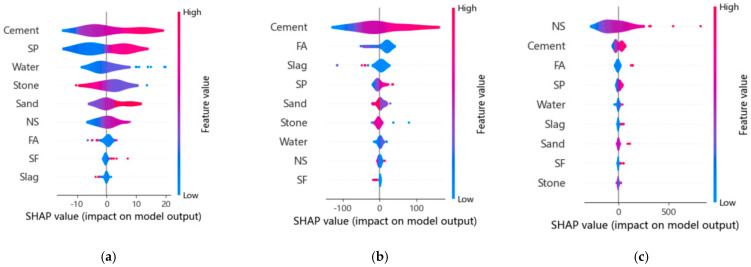
SHAP value results of the XGB model: (**a**) Compressive strength; (**b**) Carbon emissions; (**c**) Cost.

Figure 10a analyzes the impact of 9 design variables on the compressive strength of NSC. The vertical axis represents the importance of design variables to the compressive strength of NSC, while the horizontal axis represents the distribution of SHAP values. The zero value on the horizontal axis represents the predicted average compressive strength of NSC, and the color bar indicates the variation in the values of the design variables. It can be observed that the parameter with the greatest impact on the compressive strength of NSC is cement. The factors influencing the compressive strength of NSC, in descending order, are as follows: Cement > SP > Water > Stone > Sand > NS > FA > SF > Slag.

Figure 10b analyzes the impact of 9 design variables on the carbon emissions of NSC. It can be observed that the factors influencing the carbon emissions of NSC, in descending order, are as follows: Cement > FA > Slag > SP > Sand > Stone > Water > NS > SF.

Figure 10c analyzes the impact of 9 design variables on the cost of NSC. It can be ob-served that the factors influencing the cost of NSC, in descending order, are as follows: NS > Cement > FA > SP > Water > Slag > Sand > SF > Stone.

### 5.3. Multi-Objective Optimization

**Figure 11:**
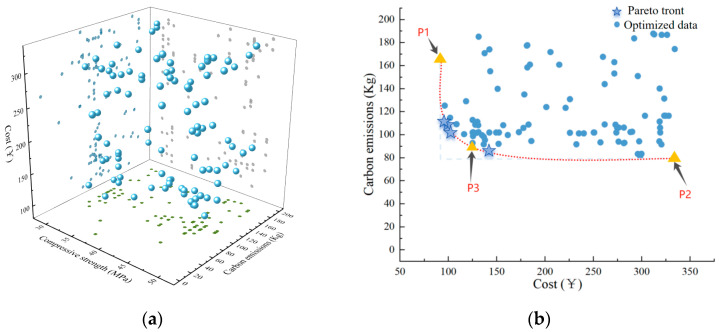
Relationship diagram of compressive strength, cost, and carbon emissions, and pareto front diagram: (**a**) Relationship diagram; (**b**) Front points diagram.

From Figure 11, it can be observed that the Pareto front is relatively evenly distributed. As the concrete strength increases, both the cost and carbon emissions increase accordingly. The maximum strength value can reach 49 MPa, with corresponding cost and carbon emissions of 156.8 yuan/m^3^ and 108.16 kg, respectively. The minimum cost can be as low as 92.18 yuan/m^3^, with corresponding strength and carbon emissions of 36 MPa and 165.69 kg, respectively. The minimum carbon emissions can be as low as 80.14 kg, with corresponding strength and cost of 42 MPa and 334.23 yuan/m^3^, respectively. This indicates that high strength is contradictory to low cost and low carbon emissions, as the use of more cementitious materials provides strength but simultaneously increases cost and carbon emissions.

As mentioned above, several sets of optimal solutions are generated instead of a single optimal solution. In this case, the results vary depending on different engineering requirements. Therefore, 100 results are obtained through multi-objective optimization for researchers to choose from.

In this study, three key points are selected to guide practical engineering applications (Table 8). Among them, point P1 is located at the far left of the Pareto front and is used to determine the optimal mix design from the perspective of economic efficiency of concrete. Considering environmental benefits, point P2 at the bottom of the Pareto front is used to determine the optimal mix design. Additionally, at the intersection of the lines passing through P1 and P2 and parallel to the XY axis, a utopia point is formed, which satisfies the lowest cost and the lowest carbon emissions. In this case, P3 is the ideal point closest to the utopia point, serving as the basis for considering the trade-off between cost and carbon emissions.

### 5.4. Limitations

It should be noted that, due to the limited diversity of the dataset used in this study, the model is most suitable for scenarios with characteristics similar to the training data. Expanding the dataset to include more diverse nano-silica concrete mixtures and incorporating transfer learning techniques would further improve the generalization capability of the model. Moreover, it should be emphasized that the cost and carbon emission values reported in this study are provided for reference purposes only and are primarily intended to illustrate the algorithmic framework. Significant variations may occur across different regions and markets.

## 6. Conclusions

(1) A predictive model for the compressive strength of nano-silica concrete was developed using four artificial intelligence algorithms, with Bayesian applied for hyperparameter optimization. Among them, the XGBoost model exhibited the best performance, achieving R^2^ = 0.99, RMSE = 1.80 MPa, MAE = 0.68 MPa, MAPE = 2.48%.
(2) Feature importance analysis revealed that nano-silica content was highly correlated with compressive strength (0.82) and cost (0.85), while showing a moderate correlation with carbon emissions (0.78).
(3) By integrating XGBoost with NSGA-II, a multi-objective optimization framework was established, considering strength, cost, and carbon emissions. A Pareto frontier selection approach was proposed, enabling the identification of mix designs with maximum strength, minimum cost, or minimum carbon emissions. This method reduces the number of required experiments, enhances design efficiency, and supports sustainable concrete production.

## Figures and Tables

**Table 1:** Compressive strength database statistical information table.

Input/Output	Mean	Maximum	Minimum
Cement kg/m^3^	349.31	630	80
Fly ash kg/m^3^	86.84	685	0
Slag kg/m^3^	79.36	1134	0
Nano silica(NS) kg/m^3^	7.46	60	0
silica fume (SF) kg/m^3^	2.51	60	0
Water kg/m^3^	216.64	1007.55	90
Superplasticizer (SP) kg/m^3^	3.67	21.38	0
Stone kg/m^3^	1005.65	1278	654
Sand kg/m^3^	948.18	9320	472
Compressive Strength(CS) MPa	39.34	95.25	2.30
Carbon emission (CO_2_) kg	273.18	504.88	72.22
Cost (¥)	324.52	1503.63	86.46

**Table 4:** Carbon emission calculation parameters.

Carbon Emission Components	Carbon Emission Sources	Calculation Parameters	
		Notation Symbols	Content
Embodied carbon emissions from raw materials per unit of concrete product	Embodied carbon emissions of raw materials	${EF}_{i,h}$	Unit carbon emissions of raw materials such as cement, nano-silica, superplasticizer, sand, and aggregate
		$M_{i}$	Mix proportion per cubic meter of concrete
	Carbon emissions from fuel used in raw material transportation	$H_{i}$	Transportation distance of raw materials
		/	Transportation mode of raw materials
Production of carbon emissions per unit of concrete product	Carbon emissions from energy consumption in concrete production	$E_{d}$	Total electricity consumption of the enterprise during a specific Production period
		N	Total concrete production of the enterprise during a specific production period

**Table 5:** Survey data on carbon emissions of raw materials.

Type of Raw Materials	Unit Raw Material Production Carbon Emissions ${EF}_{i}/kg\;{kg}^{-1}$ **[39,40]**	Transportation Mode	$\mathrm{Transportation}\;\mathrm{Distance}\;H_{i}/km$
Cement	1.405	Land transportation	60
Fly ash	0	Land transportation	0
Slag	0	Land transportation	0
Nano-silica	0.0139	Land transportation	25
water	0	Land transportation	0
Superplasticizer	0.02849	Land transportation	15
Stone	0.00312	Land transportation	20
Sand	0.0012	Land transportation	20

**Table 6:** Concrete production survey data.

Data Type (Taking a Concrete Plant as an Example)	Numerical Value
$\mathrm{Electricity}\;\mathrm{consumption}/E_{d}\cdot kWh^{-1}$	2,502,679
Production capacity N/10 kt	74.34

**Table 7:** Statistical performance metrics of each machine learning model.

Models	$R^{2}$	MAPE	MAE (MPa)	RMSE (MPa)
CatBoost	0.96	4.9	2.79	2.25
XGBoost	0.99	2.48	0.68	1.8
RF	0.95	5.63	3.91	3.2
AdaBoost	0.97	3.77	1.7	4.46

**Table 8:** Multi-objective mix proportion table based on compressive strength, carbon emissions, and cost.

Parameter	Unit	Lowest-Cost P1	Lowest Carbon Emission P2	Trade-Off Scheme
cement	kg/m^3^	200.81	80.58	112.66
fly ash	kg/m^3^	36.53	75.30	93.48
slag	kg/m^3^	0.00	311.45	0.44
nano-silica	kg/m^3^	6.69	3.68	31.11
silica fume	kg/m^3^	0.00	26.74	8.65
water	kg/m^3^	90.01	195.98	212.00
superplasticizer	kg/m^3^	2.09	3.45	93.84
coarse aggregate	kg/m^3^	1014.00	1184.00	743.50
fine aggregate	kg/m^3^	771.76	680.09	741.56
age	Day	28	28	28
compressive strength	MPa	36.00	42.00	30.00
carbon emission	kg/m^3^	165.69	80.14	91.57
cost	¥	92.2	334.2	136.6

## Data Availability

Data will be made available on request.
